# SH3BGRL3 binds to myosin 1c in a calcium dependent manner and modulates migration in the MDA-MB-231 cell line

**DOI:** 10.1186/s12860-021-00379-1

**Published:** 2021-08-11

**Authors:** Filippo Di Pisa, Elisa Pesenti, Maria Bono, Andrea N. Mazzarello, Cinzia Bernardi, Michael P. Lisanti, Giovanni Renzone, Andrea Scaloni, Ermanno Ciccone, Franco Fais, Silvia Bruno, Paolo Scartezzini, Fabio Ghiotto

**Affiliations:** 1 Department of Experimental Medicine, University of Genoa, 16132 Genoa, Italy; 2 Translational Medicine, School of Science, Engineering and Environment (SEE), University of Salford, Greater Manchester, UK; 3 Wellcome Trust Centre for Cell Biology, Institute of Cell Biology, University of Edinburgh, Edinburgh, UK; 4 Karches Center for Oncology Research, The Feinstein Institute for Medical Research, Northwell Health, Manhasset, NY 11030 USA; 5 Molecular Pathology Unit, IRCCS Policlinico San Martino, 16132 Genoa, Italy; 6 Proteomics and Mass Spectrometry Laboratory, ISPAAM-National Research Council, 80147 Naples, Italy; 7 Ente Ospedaliero Ospedali Galliera, 16128 Genoa, Italy

**Keywords:** SH3BGRL3, Myosin 1c, IQ domain, Cell migration

## Abstract

**Background:**

The human *SH3 domain Binding Glutamic acid Rich Like 3* (*SH3BGRL3*) gene is highly conserved in phylogeny and widely expressed in human tissues. However, its function is largely undetermined. The protein was found to be overexpressed in several tumors, and recent work suggested a possible relationship with EGFR family members.

We aimed at further highlighting on these issues and investigated SH3BGRL3 molecular interactions and its role in cellular migration ability.

**Results:**

We first engineered the ErbB2-overexpressing SKBR3 cells to express exogenous SH3BGRL3, as well as wild type Myo1c or different deletion mutants. Confocal microscopy analysis indicated that SH3BGRL3 co-localized with Myo1c and ErbB2 at plasma membranes. However, co-immunoprecipitation assays and mass spectrometry demonstrated that SH3BGRL3 did not directly bind ErbB2, but specifically recognized Myo1c, on its IQ-bearing neck region. Importantly, the interaction with Myo1c was Ca^2+^-dependent.

A role for SH3BGRL3 in cell migration was also assessed, as RNA interference of SH3BGRL3 in MDA-MB-231 cells, used as a classical migration model, remarkably impaired the migration ability of these cells. On the other side, its over-expression increased cell motility.

**Conclusion:**

The results of this study provide insights for the formulation of novel hypotheses on the putative role of SH3BGRL3 protein in the regulation of myosin-cytoskeleton dialog and in cell migration. It could be envisaged the SH3BGRL3-Myo1c interaction as a regulation mechanism for cytoskeleton dynamics. It is well known that, at low Ca^2+^ concentrations, the IQ domains of Myo1c are bound by calmodulin. Here we found that binding of Myo1c to SH3BGRL3 requires instead the presence of Ca^2+^. Thus, it could be hypothesized that Myo1c conformation may be modulated by Ca^2+^-driven mechanisms that involve alternative binding by calmodulin or SH3BGRL3, for the regulation of cytoskeletal activity.

**Supplementary Information:**

The online version contains supplementary material available at 10.1186/s12860-021-00379-1.

## Background

The human *SH3BGRL3* gene (SH3 Domain Binding Glutamate Rich Protein Like 3) (also named *TIP-B1*) was cloned by two independent groups [1, 2]. It belongs to a poorly characterized gene family, comprising three other members: *SH3BGR*, selectively expressed in muscle tissue [3, 4], and *SH3BGRL* [5] and *SH3BGRL2* [6], that display ubiquitous expression. The *SH3BGRL3* gene is also ubiquitously expressed, is well conserved in phylogeny and is found in species evolutionary distant from human. For example, an ortholog of *SH3BGRL3* gene is expressed in zebrafish, where it is already expressed in the early stages of embryonic development [7]. These observations indicate a high degree of evolutionary conservation of the gene and suggest an important biological role for the SH3BGRL3 protein.

In humans, *SH3BGRL3* maps to chromosome 1p34.3–35 and codes for a 93 amino acids protein whose three-dimensional structure has been resolved by X-ray crystallography [8] and nuclear magnetic resonance (NMR) [9]. SH3BGRL3 protein shows similarities with glutaredoxins, although it lacks the canonical sequence of the corresponding redox active sites, and thus is devoid of enzymatic activity [1]. In addition, unlike the other proteins of the family, it does not have a canonical SH3 (Src Homology 3) binding domain. Indeed, while SH3BGR, SH3BGRL and SH3BGRL2 proteins contain the PLPPQIF sequence, which corresponds to the SH3 consensus sequence PXXPQ(L/I)(Y/F), SH3BGRL3 displays only a PPQIV sequence [1, 6].

Very little information is available on its function and its molecular interactors. In TNF-treated fibroblasts it was characterized as having anti-apoptotic function [10]. All other studies were carried out in tumor models. Increased *SH3BGRL3* expression was found in several tumors, such as lung adenocarcinoma [11], bladder [12] and kidney [13] cancer. Protein level in the urine of urothelial carcinoma patients was positively associated with tumor high grade and invasiveness [12]. High *SH3BGRL3* expression in no muscle-invasive bladder cancer significantly correlated with increased risk of progression [12]. The latter study also demonstrated a positive association between the amount of *SH3BGRL3 gene* expression achieved in the cells from a *SH3BGRL3*-transfected cell line and the migration capacity of the cells in vitro, supporting a role for this gene in metastasis formation and tumor aggressiveness. Also, a correlation was found between high expression of *SH3BGRL3* and tumor progression in kidney clear cell carcinoma (KIRC) [13]; moreover, *SH3BGRL3* knockdown inhibited KIRC cells proliferation, migration and invasion in vitro, and suppressed tumor growth and metastasis in vivo [13]. Interestingly, the authors of this study also observed that the SH3BGRL3 protein may interact with EGFR and inhibit its degradation thus enhancing the EGF-induced AKT signaling [13]. Likewise, an analysis of the interactome of proteins of the EGFR family showed that the SH3BGRL3 protein can interact with ErbB1/EGFR and ErbB2/HER2 [14], both of which are important players in carcinogenesis [15]. Specifically, the SH3BGRL3 protein could bind to the intracellular portion of ErbB1 at the phosphorylated Tyr891 residue, and of ErbB2 at the phosphorylated Tyr923 and Tyr1196 residues [14]. However, protein structure analysis indicated that the SH3BGRL3 protein lacks the SH2 domain [1, 6, 8] which is required for the direct docking to phosphorylated Tyr residues. This result implies that SH3BGRL3 might only indirectly interact with phosphorylated EGFR through other adaptor proteins.

In this complex and still uncertain scenario, the aim of this work was that of obtaining new insights into the interactions and function of SH3BGRL3. We found that the SH3BGRL3 protein interacts with the IQ-bearing neck region of the motor protein Myo1c, and that SH3BGRL3, Myo1c and ErbB2 co-localize at plasma membrane, possibly in membrane ruffles. We also observed that SH3BGRL3 protein levels play a role in cell migration ability.

## Results

### SH3BGRL3 protein binds to myosin 1c but not ErbB2 in SKBR3 cells

**Fig. 1:**
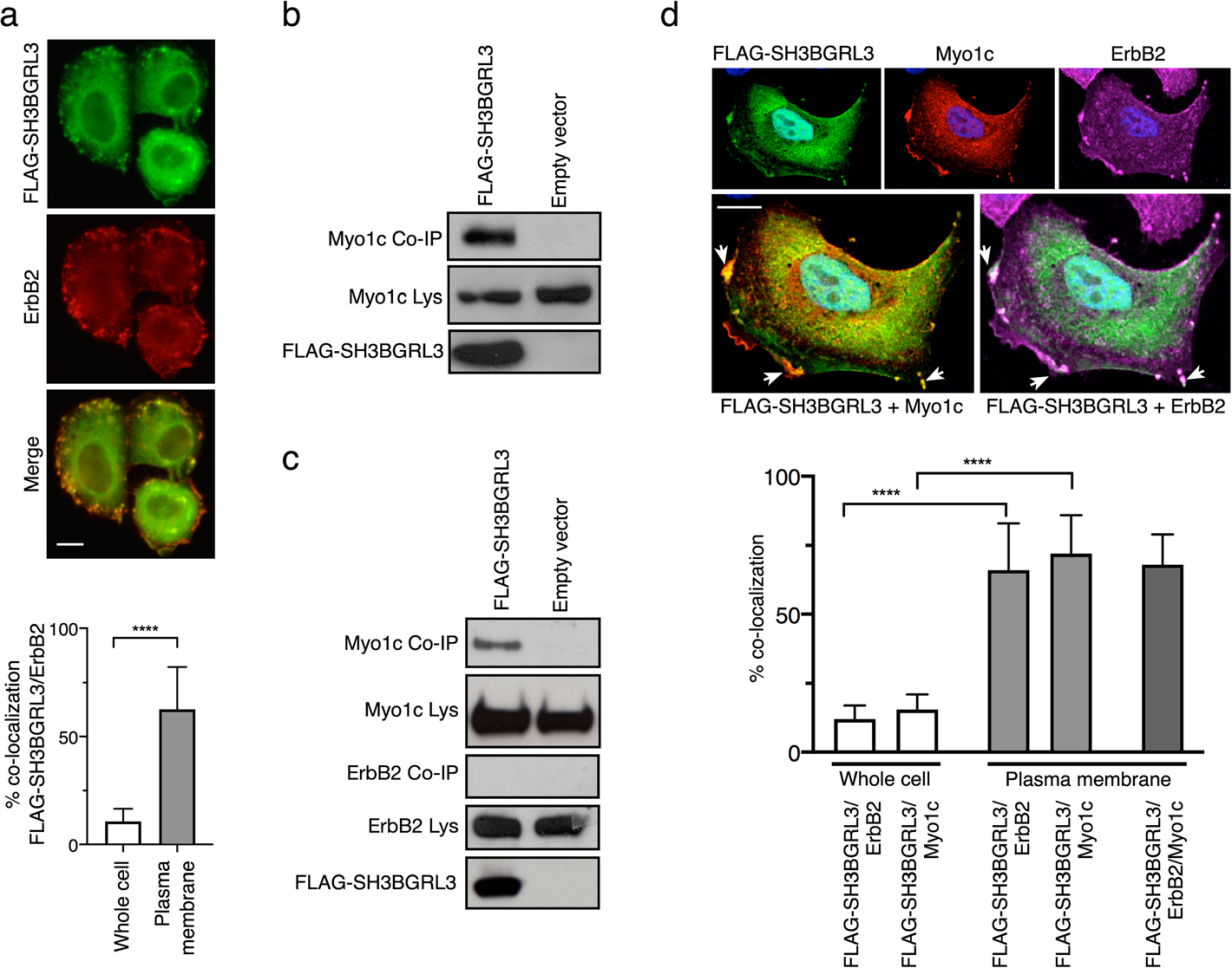
SH3BGRL3 colocalizes with both ErbB2 and Myo1c but interacts solely with Myo1c. (**a**) Upper: representative confocal image of SKBR3 cells transfected with FLAG-SH3BGRL3 and stained with anti-FLAG and anti-ErbB2 mAbs. . Scale bar = 10 μm. Lower: co-localization levels expressed as % of SH3BGRL3-positive pixels that are also positive (over threshold) for ErbB2 fluorescence, evaluated either on the whole FLAG-SH3BGRL3-transfected cell (left) or on ROIs gating the plasma membrane region (right) (see Methods). (**b**) WB analysis with anti-Myo1c antibody of Co-IP (upper inset) and lysates (middle inset) from transfected cells (left lane) or from cells transfected with an empty vector (right lane). The lower inset displays the lysates revealed with an anti-FLAG antibody to verify the presence of the FLAG-SH3BGRL3 bait. (**c**) Co-IP of endogenous Myo1c and ErbB2 using FLAG-SH3BGRL3 (left lane), and the empty vector as negative control (right lane). WB analysis with anti-Myo1c antibody of Co-IP and lysates (two upper insets), WB analysis with anti-ErbB2 antibody of Co-IP and lysates (third and forth insets). The lower inset displays the lysates revealed with an anti-FLAG antibody to verify the presence of the FLAG-SH3BGRL3 bait. (**d**) Upper: representative confocal image of SKBR3 cells transfected with FLAG-SH3BGRL3 and stained with anti-FLAG, anti-Myo1c and anti-ErbB2 antibodies. Arrows point to structures with a morphology that highly resembles membrane ruffles. Scale bars = 10 μm. Lower: co-localization levels expressed as % SH3BGRL3-positive pixels that are also positive (over threshold) for Myo1c or ErbB2 fluorescence, evaluated either on the whole FLAG-SH3BGRL3-transfected cell (first two bars from the left) or on ROIs gating the plasma membrane region (two subsequent bars) (see Methods). To further evaluate whether the SH3BGRL3/ Myo1c co-localizing spots on plasma membranes were SH3BGRL3/ ErbB2 positive as well, the triple SH3BGRL3/Myo1c/ErbB2 co-localization levels were addressed by measuring the % of Myo1c/ErbB2 co-localizing pixels that also contained SH3BGRL3-positive fluorescence (last bar from left) (see Methods)

The ErbB2-positive breast cancer cell line SKBR3, a widely used model to study ErbB2 expression, was chosen to assess the co-localization of SH3BGRL3 and ErbB2 proteins by confocal microscopy. For this purpose, we transfected SKBR3 cells with a plasmid vector expressing an N-terminal flagged version of the SH3BGRL3 sequence (FLAG-SH3BGRL3). The FLAG was necessary because available anti-SH3BGRL3 antibodies displayed good sensitivity for the denaturated protein, but showed very poor binding in the case of in situ folded protein. Thus, we had used anti-FLAG antibodies to detect SH3BGRL3 with proper sensitivity. Staining of the transfected cells with anti-ErbB2 and anti-FLAG mAbs showed a relevant degree of co-localization which was mostly confined to membrane structures (Fig. 1a).

We then investigated the SH3BGRL3-ErbB2 interaction by co-immunoprecipitation (Co-IP); we set up dedicated experiments using both FLAG-SH3BGRL3 and ErbB2 as bait. However, no co-precipitation of the two molecules could be detected (data not shown), at variance with previous literature data [14].

In order to investigate additional proteins that could interact with SH3BGRL3, including those potentially capable of mediating an interaction with ErbB2, we used the following strategy. Lysates of SKBR3 cells, transfected either with FLAG-SH3BGRL3 or with the empty vector (control), were exposed to an anti-FLAG-coupled resin and the bound proteins were subsequently eluted and analyzed by SDS-PAGE. Three protein bands were present in the co-immunoprecipitate of FLAG-SH3BGRL3-transfected cells and not in those from the control cells. (Additional File 1). Mass spectrometry analyses of the three bands identified the following proteins as potentially capable of interacting with SH3BGRL3: i) myosin 1c (Myo1c), which is a non-conventional monomeric myosin; ii) adseverin (also known as scinderin), which is an F-actin severing protein; iii) actin (Additional File 2). All these proteins have a role in the cytoskeleton turn-over [16–18]. In particular, Myo1c is an interesting complex protein, which harbors several regulatory domains. It is a monomeric motor protein [19], expressed in all eukaryotic cells and localized primarily at the actin-rich cellular periphery along the plasma membrane [20]. Myo1c seems to exert an important role in the communication between the cell and its environment by transducing signals from cell membrane receptors to the cytoskeleton [16]. Thus, we focused on the association between SH3BGRL3 and Myo1c proteins. To validate the interaction between SH3BGRL3 and Myo1c, a co-immunoprecipitation analysis was carried out in SKBR3 cells transfected with the FLAG-SH3BGRL3 construct as above, except that the material eluted from the resin was analyzed by WB using either anti Myo1c (Fig. 1b) or anti ErbB2 antibodies (Fig. 1c). As evident from the figure, Myo1c, but not ErbB2, was present in the immunoprecipitates with FLAG-SH3BGRL3 antibodies, thus validating our previous unsuccessful attempts of co-immunprecipitating both FLAG-SH3BGRL3 and ErbB2 from the same cell line. Confocal microscopy analysis of the intracellular distribution of FLAG-SH3BGRL3, Myo1c and ErbB2 in SKBR3 cells indicated a remarkable co-localization of the three proteins, which was primarily confined to the plasma membrane, in structures that morphologically highly resemble membrane ruffles (Fig. 1d).

### SH3BGRL3 protein binds the neck region of Myo1c in a Ca^2+^ dependent manner

Myo1c shares a common structure with all the other members of the myosin superfamily. This is comprised of three domains, namely a ‘head’ region, that hydrolyzes ATP and interacts with actin, a ‘neck’ region, that contains three IQ calmodulin-binding domains plus a post-IQ portion [21], and a ‘tail’ region, which includes a pleckstrin-homology domain that allows Myo1c to interact with the cell membrane [22].

**Fig. 2:**
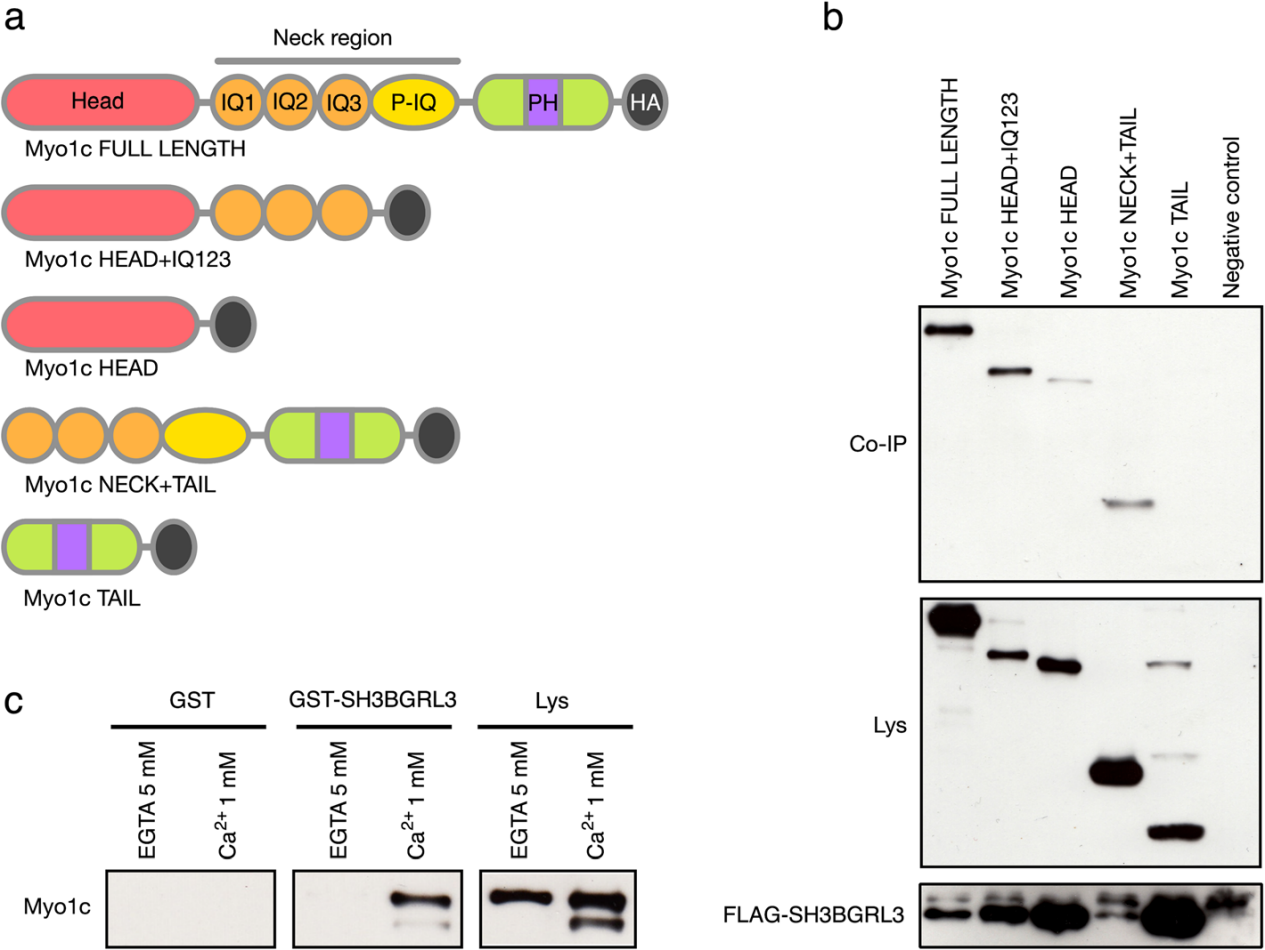
SH3BGRL3 binds to the neck region of Myo1c in a calcium dependent manner. (**a**) Diagram of the wild type Myo1c domains and of the deletion mutants used for the Co-IP experiments. (**b**) SKBR3 cells were co-transfected with wild type and deletion mutant Myo1c constructs, each carrying an HA tag, and FLAG-SH3BGRL3 construct. FLAG-SH3BGRL3 and Myo1c variants were co-immunoprecipitated using anti-FLAG conjugated resin, and Myo1c variants revealed using an anti-HA antibody. Co-IP of a lysate from SKBR3 cells not transfected with any Myo1c construct was used as negative control. (**c**) Endogenous Myo1c was pulled-down using GST-SHBGRL3 fusion protein or GST alone bound to Sepharose-Glutathione 4B, in presence of Ca^2+^ or EGTA and revealed with an anti-Myo1c antibody. P-IQ: Post-IQ domain; PH: pleckstrin-homology domain; HA: HA tag

In order to further characterize the interaction between SH3BGRL3 and Myo1c, we designed and expressed plasmid constructs coding for the wild type Myo1c (Myo1c FULL LENGTH) and for protein deletion mutants from which one or more domains were missing (Fig. 2a). The following mutants were used: i) Myo1c HEAD+IQ123, a mutant that was comprised of the gene segments encoding the head region and the three IQ domains ii) Myo1c HEAD, made by the segment encoding the head region alone iii) Myo1c NECK+TAIL that encoded three IQ domains plus the region between the IQ domains and the tail plus the tail itself iv) Myo1c TAIL, encoding for the tail alone. All the constructs carried an HA segment encoding for the HA epitope located at the C-terminus of the expressed proteins (Fig. 2a). SKBR3 cells were co-transfected with these constructs together with the FLAG-SH3BGRL3 vector. Co-IP experiments were then performed on the lysates from these cells with an anti-FLAG conjugated resin using the same strategy described above. Evidence for binding to SH3BGRL3 was obtained only in the WB of lysates from cells transfected with constructs containing the IQ domains (Fig. 2b). This indicates that the SH3BGRL3 protein interacts with the neck region of Myo1c. The same Myo1c region has been reported to bind calmodulin (CaM), a Ca^2+^ binding protein [21]. Since this binding occurs only in the absence of calcium [23], we asked the question of whether SH3BGRL3-Myo1c interactions were influenced by the absence or the presence of Ca^2+^. To this end, we made a construct to express the GST-SH3BGRL3 fusion protein in bacteria. The purified GST-SH3BGRL3 fusion protein bound to Sepharose-Glutathione 4B was used to perform pull-down experiments from lysates of SKBR3 in the presence/absence of Ca^2+^ or of EGTA. As evident from Fig. 2c, GST-SH3BGRL3-Myo1c interactions required the presence of Ca^2+^.

### SH3BGRL3 does not bind the IQ domains of Myo1c only

To further corroborate the notion of a direct binding of SH3BGRL3 to IQ domain-harboring proteins, we studied the interaction of SH3BGRL3 with GAP43 (neuromodulin), a small protein that contains a single IQ domain [24]. This protein was selected since, unlike the Myo1c protein, can be obtained easily in a recombinant, purified form in vitro.

**Fig. 3:**
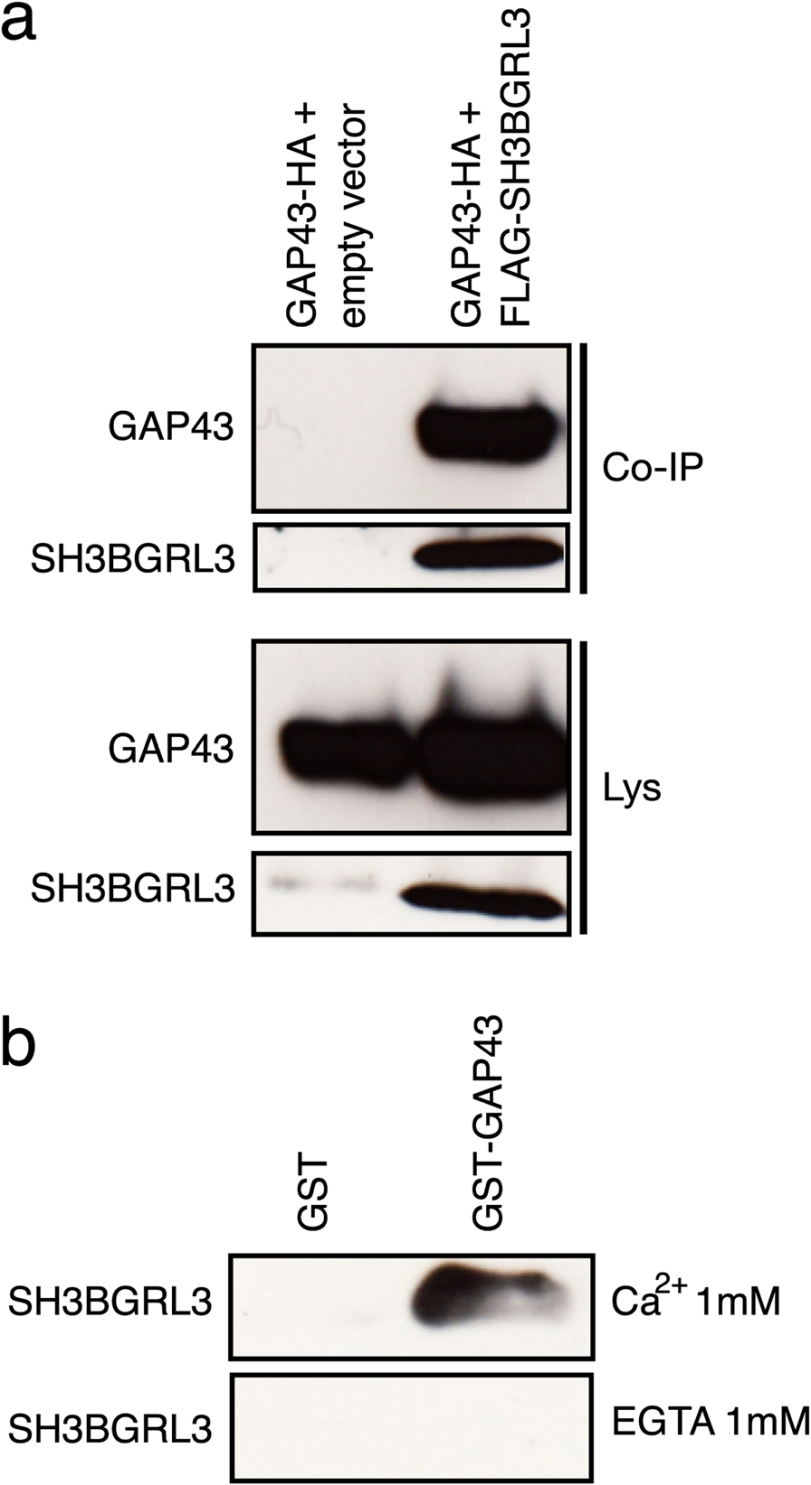
SH3BGRL3 binds to GAP43 protein in presence of Ca^2+^. (**a**) SKBR3 cells were co-transfected with a construct coding for HA-tagged GAP43 and a construct coding for FLAG-SH3BGRL3 or the empty vector. FLAG-SH3BGRL3 and GAP43 were co-immunoprecipitated using an anti-FLAG conjugated resin and GAP43 revealed using an anti-HA antibody. (**b**) Purified histidine tagged SH3BGRL3 was pulled-down using GST-GAP43 fusion protein or GST alone bound to Sepharose-Glutathione 4B in presence of Ca^2+^ or EGTA. SH3BGRL3 was revealed using an anti-SH3BGRL3 antibody

A HA-tagged *GAP43* gene was cloned and expressed into an expression vector. SKBR3 cells were then co-transfected with both a HA-tagged GAP43 and a FLAG-tagged SH3BGRL3 vector (or an empty vector as negative control). After co-immunoprecipitation of the cell lysates using an anti-FLAG coupled resin, GAP43 was found to be consistently present in the immune-precipitates (Fig. 3a). To assess whether there is a direct SH3BGRL3-GAP43 interaction or else this interaction is mediated by other proteins present within a macromolecular complex in the cell lysates, a GST-GAP43 fusion protein was expressed in bacteria. The purified GST-GAP43 protein was then coupled to Sepharose-Glutathione 4B and used for pull-down experiments of purified histidine-tagged SH3BGRL3 protein in presence of Ca^2+^ or of EGTA. The results demonstrated that SH3BGRL3 is capable of binding to GAP43 directly, and, interestingly, only in the presence of Ca^2+^ (Fig. 3b).

### SH3BGRL3 plays a role in cellular migration ability

**Fig. 4:**
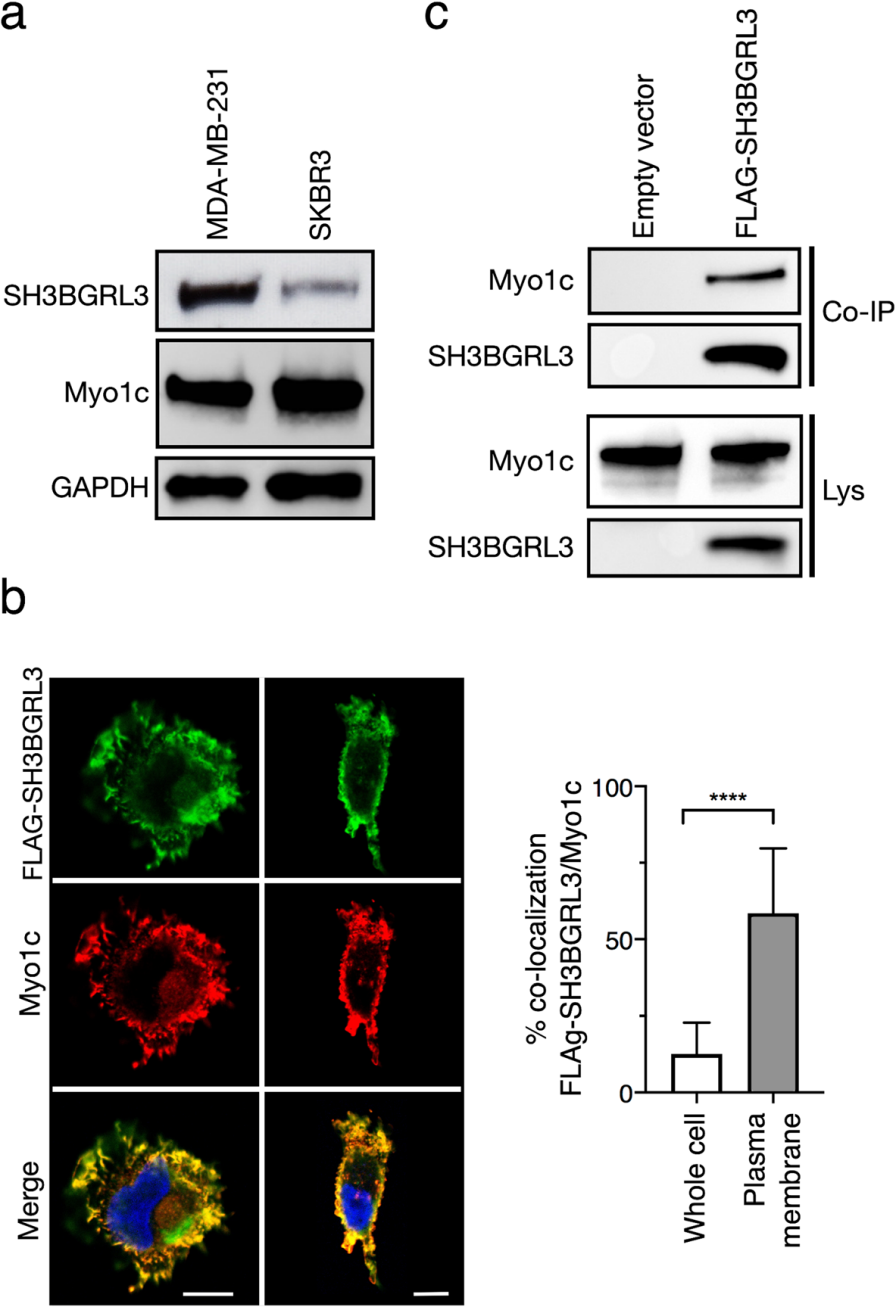
SH3BGRL3 and Myo1c co-localize and interact also in MDA-MB-231 cells. (**a**) Western blot of MDA-MB-231 and SKBR3 lysates using anti-SH3BGRL3, anti-Myo1c and anti-GAPDH antibodies. (**b**) Left: two representative confocal microscopy images of FLAG-SH3BGRL3 transfected MDA-MB-231 cells stained with anti-FLAG and anti-Myo1c antibodies. Scale bar = 10 μm. Right: co-localization levels expressed as % SH3BGRL3-positive pixels that were also positive (over threshold) for Myo1c fluorescence, evaluated either on the whole FLAG-SH3BGRL3-transfected cell (left) or on ROIs gating the plasma membrane region (right). (**c**) FLAG-SH3BGRL3 and Myo1c were co-immunoprecipitated from lysates of FLAG-SH3BGRL3 transfected MDA-MB-231 cells and revealed with anti-FLAG and anti-Myo1c antibodies. Scale bar = 10 μm

Myo1c is involved in several cellular functions such as generation of cell membrane tension [25], lipid raft recycling to control cell spreading and migration [26], and G-actin transport at the leading edge of migrating cells [27]. Based on these premises, the role of the SH3BGRL3-Myo1c interaction in cell migration was investigated using the MDA-MB-231 cell line, which represents a classical model for studies of this type. To this end, SH3BGRL3 protein was first demonstrated to be present in the MDA-MB-231 cell line as could be assessed by WB (Fig. 4a); then, the co-localization of SH3BGRL3 with Myo1c in plasma membrane structures was confirmed by confocal microscopy in these cells too (Fig. 4b); third, the SH3BGRL3-Myo1c interactions in MDA-MB-231 were documented by co-immunoprecipitation experiments using FLAG-SH3BGL3 transfected MDA-MB-231 cells utilizing the same procedure described above (Fig. 4c).

**Fig. 5:**
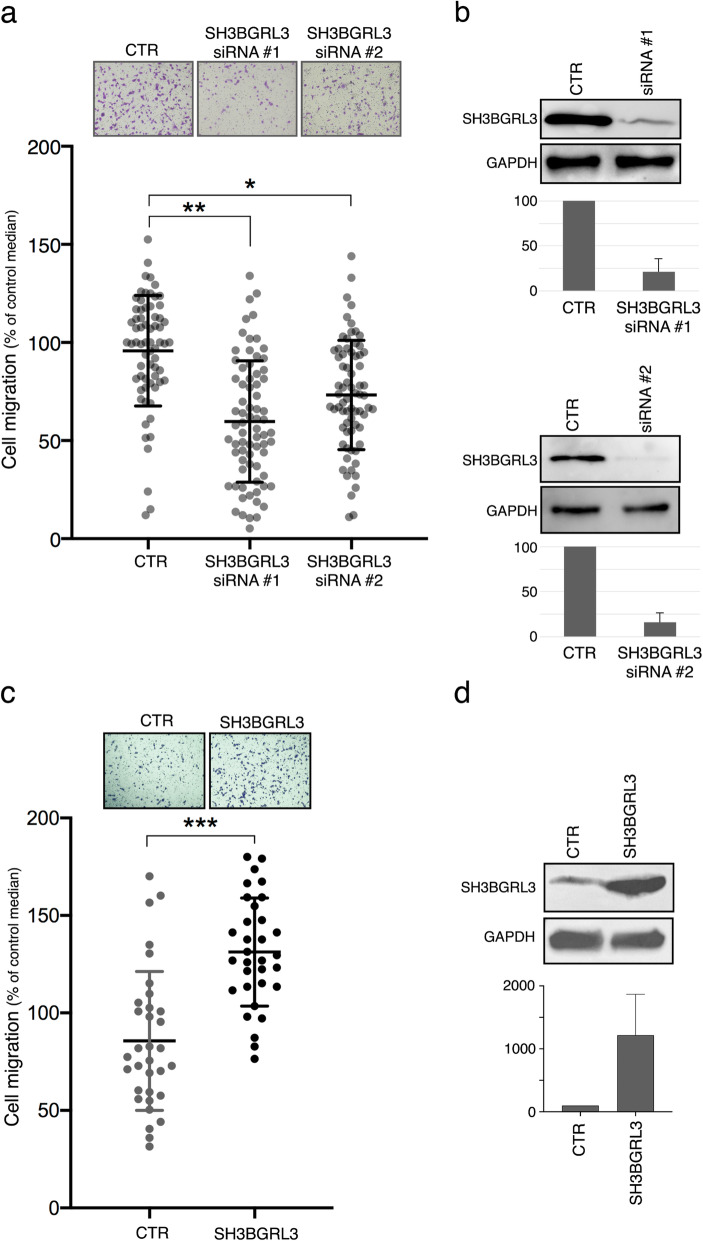
Modulation of SH3BGRL3 expression impacts on the migration features of MDA-MB-231 cells. (**a**) Upper: Representative microscopic images of Boyden chamber fields of migrated MDA-MB-231 cells transfected with control scrambled siRNA (CTR) or with SH3BGRL3 specific siRNAs (siRNA #1 or siRNA #2). Lower: Quantification of the migration ability of the same cells as above. Three independent migration experiments were carried out, in duplicate or triplicate. Each chamber slide was divided in nine fields and cell counts/field reported in the graph. For each chamber slide of the scramble controls, the median cell counts/field was set to 100, and data of SH3BGRL3 siRNAs were normalized to their respective scramble control. Field counts for all experiments are gathered in this graph, mean ± SD are reported and unpaired t-test was used for evaluating statistical difference significance. (**b**) Western blots showing the silencing efficiency of the two SH3BGRL3 specific siRNAs. Below are the SH3BGRL3/GAPDH ratios, normalized to the scramble control ratio, which was set to 100. **c**) Upper: Representative microscopic images of Boyden chamber fields of migrated MDA-MB-231 cells subjected to lentiviral infection of either control (CTR) or SH3BGRL3 vectors. Lower: Quantification of the migration ability of the same cells as above in two independent migration experiments, each carried out in duplicate. Cell counts and statistical analysis were performed as detailed in a). (**d**) Western blots showing the efficiency of the over-expression system. Below are the SH3BGRL3/GAPDH ratios, normalized to the control ratio, which was set to 100

Thus, MDA-MB-231 cells were used to study how modulation of SH3BGRL3 expression affects cell migration. MDA-MB-231 cells were first transfected with either a control siRNA or one of two different siRNAs specific for the SH3BGRL3 sequence. Results from migration assays carried out using Boyden chambers indicated that downregulation of SH3BGRL3 expression results in a significant decrease of the migration capacity, for either siRNA (Fig. 5a). Figure 5b displays the quantification of the SH3BGL3 down-regulation observed for the two siRNAs, together with a representative WB.

On the other side, to test whether over-expression of SH3BGRL3 could increase cell migration ability, we infected MDA-MB-231 cells with lentivirus particles carrying the *SH3BGRL3* lentiviral expression plasmid (or the same empty vector as a control). Cell migration in Boyden chambers resulted to be significantly increased by SH3BGRL3 over-expression (Fig. 5c), which was assessed by western-blot (Fig. 5d).

## Discussion

This study demonstrates that SH3BGRL3 interacts with the motor protein Myo1c in two different cell lines (SKBR3 and MDA-MB-231). In SKBR3 cells, these proteins co-localize in plasma membrane regions resembling membrane ruffles, where also the ErbB2 protein co-localizes with them. Moreover, silencing of SH3BGRL3 expression resulted in a decrease of cell migration capacities of the classical cell migration model MDA-MB-231, implying that SH3BGRL3-Myo1c interactions are crucial for cell locomotion. Finally, it was also demonstrated that SH3BGRL3 binds to the neck region of Myo1c, which is composed of three IQ domains. The specificity for the IQ domains was confirmed further by the capacity of SH3BGRL3 to bind to another protein, namely GAP43, which harbors a single IQ domain. The finding that SH3BGRL3 binds with the same modality to another IQ domain-bearing protein with a functional role different from that of Myo1c, suggests that SH3BGRL3 may interact with several proteins with IQ domains and serve different cellular functions. SH3BGRL3 was able to bind to the IQ domains of Myo1c - as well as to that of GAP43 - only in presence of Ca^2+^, whereas calcium chelators (e.g. EGTA) hindered the binding, possibly suggesting that Ca^2+^ levels exert a regulatory function on Myo1c. At low Ca^2+^ concentrations, the IQ domains of Myo1c are bound by calmodulin and calmodulin-like proteins (CaMs) [21]. This binding increases the stability of the Myo1c ‘neck region’, which is crucial for transducing the tension generated by the motor domain to the ‘tail region’. When the cellular Ca^2+^ concentration rises, CaMs dissociate from the IQ domains and the Myo1c ‘neck region’ becomes more flexible [21]. In this form, Myo1c is likely to become capable of being bound by SH3BGRL3. Therefore, it could be hypothesized that the conformation and the stiffness of the Myo1c ‘neck region’ may be modulated by Ca^2+^-driven mechanisms that cause the alternative binding of Myo1c by either calmodulin or SH3BGRL3. This may represent a mode of regulating cytoskeletal activity, which deserves further experimental testing.

Recently it was shown in urothelial carcinoma cells that SH3BGRL3 can only indirectly interact with EGFR following stimulation of the cells with EGF for 10–30 min and that this interaction occurs through Grb2 [12]. This observation may explain why, in our experimental setting, SH3BGRL3 and ErbB2 could not be co-immunoprecipitated and why a direct binding of SH3BGRL3 (that indeed does not harbor an SH2 domain) to ErbB2 was not detected. The interaction between Grb2 and SH3BGRL3 was suggested to occur through the SH3BGRL3 proline-rich motif [12]. However, SH3BGRL3 does not have a canonical SH3-binding domain [6], which is required for binding to the SH3 domain on Grb2 [28], leaving open the question of how these two proteins interact. Based on these considerations, literature data, and our own findings, it could be envisaged that Myo1c may transfer the signal triggered by ErbB2 to the actin cytoskeleton by means of a protein complex that includes Grb2 and SH3BGRL3 as two of multiple ‘adaptor proteins’.

A better understanding of the modulation role of SH3BGRL3, and particularly of the mutual relationship of this protein with ErbB2, is pivotal, given that ErbB2 is known to regulate critical cellular processes, including cell migration [29]. Indeed, Myo1c was recently found to be critical for cell migration in glioblastoma and endothelial cells. The migratory capacities of the malignant cells can also dictate their mode of spreading [27, 30]. Herein, we present evidence that SH3BGRL3 may be crucial for cell migration ability, since a substantially decreased migration capacity of MDA-MB-231 cells was observed following down-regulation of SH3BGRL3. Conversely, over-expression of SH3BGRL3 was found to enhance the migration capacity of MDA-MB-231 cells, as also observed in other cells, such as urothelial [12] and kidney [13] carcinoma cells. Altogether, these results provide insights for the formulation of novel hypotheses on the putative role of SH3BGRL3 protein in cell migration and regulation of myosin-cytoskeleton dialog.

We are aware that our data indicate that SH3BGRL3 is involved in cell migration, but do not directly demonstrate a role for the SH3BGRL3-Myo1c protein complex. Nevertheless, given that SH3BGRL3 and Myo1c mainly interact at plasma membrane structures recalling protrusions and pseudopodia involved in cellular movements, we may reason that the interaction between SH3BGRL3 and Myo1c plays a role in cellular migration.

The two cell lines used in the present study are breast cancer cells. However, this is simply coincidental. To explore the putative interaction of SH3BGRL3 with ErbB2, we chose SKBR3 cells because of their high ErbB2 expression. To analyze the effects of SH3BGRL3 modulation on cell motility we chose MDA-MB-231 cells because they are a well-known model in vitro cell migration. Nevertheless, it is of note that SH3BGRL3 might be relevant in this type of tumor, according to data from the Human Protein Atlas that show a very high SH3BGRL3 protein expression in breast cancer tissue (https://www.proteinatlas.org/ENSG00000142669-SH3BGRL3/tissue).

## Conclusions

We documented by this study that the SH3BGRL3 protein interacts with the IQ-bearing neck region of the motor protein Myo1c in the presence of Ca^2+,^ and that SH3BGRL3, Myo1c and ErbB2 co-localize at plasma membrane. Down-regulated SH3BGRL3 protein levels impair cell migration ability, which is instead increased by protein over-expression. These results provide insights for the formulation of novel hypotheses on the putative role of SH3BGRL3 protein in the regulation of myosin-cytoskeleton dialog and in cell migration. It could be envisaged the SH3BGRL3-Myo1c interaction as a regulation mechanism for cytoskeleton dynamics. Interestingly, the notion of a protein complex harboring SH3BGRL3 and Myo1c as adaptor proteins may disclose a novel mechanism of EGFR signaling to the cytoskeleton. Since the IQ domains of Myo1c are bound by calmodulin at low Ca^2+^, it could be hypothesized that Myo1c conformation may be modulated by Ca^2+^-driven mechanisms that involve alternative binding by calmodulin or SH3BGRL3, for the regulation of cytoskeletal activity.

## Methods

### Plasmid constructs

Constructs were built using the In-Fusion Advantage Cloning Kit (Clontech, CA). The DNA or cDNA template was amplified using Taq Phusion© Hot Start Flex DNA Polymerase (New England Biolabs) and specific In-Fusion Cloning Primers designed to include 15 bp homologous to the ends of the linearized target vector. The PCR products were inspected on agarose gel and then purified using Exosap (Affimetryx). The recombination of the purified PCR product with the vector was performed following the manufacturer’s protocol. Vectors used were the following: pFLAG-CMV (Sigma-Aldrich) and pIRESpuro (Clontech) for eukaryotic expression, pGEX-6P-1 (GE Life Sciences) and pQE-30 (Qiagen) for bacterial expression. The screening of positive colonies was performed using DNA amplification, positive clones were amplified, and selected clones were sequenced.

### Protein identification by mass spectrometry (MS) analysis

After SDS-PAGE, selected gel bands containing proteins of interest were excised, *in-gel* alkylated with iodoacetamide, and digested with trypsin as previously reported [31]. Peptide mixtures were desalted with μZipTipC18 tips (Millipore) prior to nano-liquid chromatography (nanoLC)-electrospray ionization (ESI)-linear ion trap (LIT)-tandem (MS/MS) mass spectrometry (nanoLC-ESI-LIT-MS/MS) analysis, which was performed on a LTQ XL mass spectrometer equipped with a Proxeon nanospray source linked to an Easy-nanoLC (Thermo Fischer Scientific, USA). Peptides were separated on an Easy C18 column (100 × 0.075 mm, 3 μm), at a flow rate of 300 nl/min, using a gradient elution with acetonitrile containing 0.1% formic acid in 0.1% formic acid, as already reported [32]. Mass spectra were acquired in the range *m/z* 400–2000 and data-dependent automatic MS/MS acquisition was applied to the three most abundant ions, enabling dynamic exclusion with repeat count 2 and exclusion duration 60 s. Mass isolation window and collision energy for peptide fragmentations were set to *m/z* 3 and 35%, respectively. Raw data from nanoLC-ESI-LIT-MS/MS analysis were searched by MASCOT v2.6.1 (Matrix Science, UK) against a UniProtKB repository with *H. sapiens* taxonomy filter (20,359 sequences, 10/2019). The following parameters were used for protein identification: mass tolerance values of 1.8 Da and 0.8 Da for precursor and fragment ions, respectively; trypsin as proteolytic enzyme with maximum missed-cleavage sites of 2; Cys carbamidomethylation as fixed modification, Met oxidation and Gln- > PyroGlu formation as variable modifications. Protein candidates with at least 2 significantly matched peptide sequences (expectation value < 0.05) with ion score > 30 were further considered for definitive protein assignment after manual spectra visualization and verification. Information on relative protein abundance in each band was derived from emPAI score values [33].

### Protein production in bacteria

Codon Plus Competent Cells (Agilent Technology) were transformed with the construct of interest. Colonies were screened for protein production and a productive colony was grown in LB + Ampicillin. Protein expression was induced with 0.1 mM IPTG for 3 h, at 37 °C. Bacteria were harvested and lysed with a sonicator (4 repetitions of 10 s each), and the recombinant protein was purified from the lysate using Sepharose-Glutathione 4B (GE Healthcare) for GST-fusion proteins cloned into pGEX-6P-1 vector, or Ni-NTA Agarose beads (Qiagen) for His-tagged protein cloned into pQE30 vector. After incubation with the resin at 4 °C, overnight, the protein was analyzed by SDS-PAGE to evaluate the molecular mass, and stored at 4 °C.

### Cell lines, transfections and infections

SKBR3 (ATCC HTB-30), MDA-MB-231 (ATCC HTB-26), and Lenti-Pac 293Ta (GeneCopoeia) cell lines were grown in DMEM medium (Euroclone) containing 10% FBS (ThermoFisher), 1% L-Glutamine (ThermoFisher) and 1 mg/mL penicillin/streptomycin (Sigma) in 5% CO_2_ humidified atmosphere.

SKBR3 and MDA-MB-231 cell lines were transiently transfected at 70–90% of confluence with the different constructs using Lipofectamine 2000 (Thermo Fisher), according to the manufacturer’s protocol. Cells were washed and lysed 48 h after transfection.

*SH3BGRL3* silencing in MDA-MB-231 was performed using two specific SH3BGRL3 Silenced Select Pre-designed siRNA (SH3BGRL3-siRNA #1 i.d. S37912, and SH3BGRL3-siRNA #2 i.d. S37910, cat. Num. 4392420, Thermo Fisher), while a Silenced Select Pre-designed negative control siRNA (Negative control #2 cat. Num. 4390847, Thermo Fisher) was used as negative control. All siRNAs were transfected using Lipofectamine 2000 (Thermo Fisher).

Overexpression of *SH3BGRL3* in MDA-MB-231 cells was obtained using the Lenti-Pac™ HIV Expression Packaging kit (GeneCopoeia) following the manufacturer’s protocol. Briefly, 293Ta lentiviral packaging cells were transfected with pReceiver-Lv105-SH3BGRL3 vector or pReceiver-LV105 empty vector. The viral particles obtained in the supernatant were used to infect MDA-MB-231 cells. Subsequently cells were selected with 1 μg/ml puromycin (Gibco) to obtain a SH3BGRL3 over-expressing stable MDA-MB-231 cell line and a MDA-MB-231 cell line carrying the empty vector used as control.

### Cell lysis and western blotting

Cells were washed with PBS (Thermo Fisher) and lysed with a scraper in EB buffer (20 mM Hepes, 150 mM NaCl, 10% w/v glycerol, 1% w/v Triton X-100) or RIPA buffer (Sigma) added with Protease Inhibitors Cocktail (Sigma). Lysates were normalized based on the total protein amount, which was measured by the Bradford assay (Bio-Rad). Samples were run in SDS-PAGE on Mini-Protean TGX Precast Protein Gels (Bio-Rad) using Colorbust Electrophoresis Marker (Sigma-Aldrich) and Leammli reducing buffer (Bio-Rad). After electrophoresis, proteins were transferred on Hybond ECL Nitrocellulose Membrane (GE Healthcare) by Western blot technique. The primary antibodies used were: a rabbit polyclonal anti-SH3BGRL3 (Product number: HPA030848, Sigma-Aldrich), a rabbit anti-Myo1c (Prod. Num. HPA001768, Sigma-Aldrich), a rabbit anti-ErbB2 (C18, sc284 Santa Cruz), a rabbit polyclonal anti-GAPDH (Santa Cruz Biotech.), a mouse monoclonal anti β-actin (A2228 Sigma-Aldrich), a mouse monoclonal anti-FLAG (Sigma-Aldrich), a mouse monoclonal anti-HA (Prod. Num. MMS-101P, Covance). HRP-conjugated goat anti-mouse and anti-rabbit (Santa Cruz Biotech.) were used as secondary antibodies. To develop the membrane, Amersham ECL Western Blotting Detection Reagent (GE Healthcare) and Amersham Hyperfilm ECL (GE Healthcare) were used. Bands were detected using an enhanced chemiluminescence system (Alliance 6.7 WL 20 M, UVITEC, Cambridge, UK) and UV1D software (UVITEC).

### Co-IP and pull-down experiments

SKBR3 and MDA-MB-231 cells were previously transfected or co-transfected with the construct(s) of interest. After 48 h, the cells were lysed as previously described, and the corresponding proteins assayed with the Bradford assay; the same amount of total proteins from above-mentioned extracts was incubated with the anti-FLAG M2 Affinity gel resin (Sigma), previously equilibrated with EB buffer. The binding was performed overnight at 4 °C, under shaking. To remove the unbound proteins, the resin was washed 5 times with EB buffer and centrifuged. Tagged proteins were detached using 10% w/v SDS. After Co-IP, proteins were loaded on SDS-PAGE and detected using western blotting (WB).

For pull-down assays, GST-fusion proteins were bound to Sepharose-Glutathione 4B (GE Healthcare). SKBR3 cells were lysed and normalized according to the total protein amount. The same amount of proteins was incubated with GST-fusion protein or GST alone at 4 °C, overnight, under shaking. The binding was performed in EB buffer containing 1 mM CaCl_2_ or EB buffer containing 1 or 5 mM EGTA. To remove the unbound proteins, resin was washed 5 times with the appropriate buffer and centrifuged. Tagged proteins were detached using 10% w/v SDS, and proteins were loaded on SDS-PAGE and detected by WB using specific antibodies.

### Immunofluorescence and confocal microscopy

After cell transfection with pCMV-FLAG/SH3BGRL3 construct or pCMV-FLAG empty vector, SKBR3 and MDA-MB-231 cells were seeded on glasses for microscopy and fixed with 3% w/v paraformaldehyde (Sigma-Aldrich) before staining. For intracellular fluorescence, cell permeabilization was performed using 0.1% w/v Triton X-100 (Sigma-Aldrich). PBS (Thermo Fisher) added with 0.5% w/v BSA (Sigma-Aldrich) was used as washing buffer, while PBS added with 2% w/v BSA was used for saturation. Monoclonal mouse anti-FLAG (Sigma-Aldrich), rabbit anti-Myo1c (Prod. Num. HPA001768, Sigma-Aldrich), and anti-ErbB2 (Herceptin, Genentech) were used as primary antibodies, while Alexa Fluor 488 goat anti-mouse (Thermo Fisher), Alexa Fluor 546 goat anti-rabbit (Thermo Fisher) and Alexa Fluor 633 goat anti-human (Thermo Fisher) were used as secondary antibodies. DAPI (Abcam) was used for nuclear staining. Images were acquired by the SP2-AOBS confocal microscope (Leica Microsystems). Original unadjusted and uncorrected images acquired under identical optical conditions were analysed by ImageJ (version J 1.34f) algorithms for the evaluation of spatial co-localization. Regions of Interest (ROIs) on FLAG-SH3BGRL3-GFP expressing cells were used, and co-localization was expressed as the percentage of SH3BGRL3-positive pixels that were also positive for ErbB2 or Myo1c fluorescence, above a background threshold that was set by the Costes’ method [34]. Around 25 cells were analysed in each of three slides, for each experiment. To evaluate co-localization of all three fluorescences, a mask obtained by the distribution of ErbB2/Myo1c co-localizing pixels was created and used to determine the fraction of SH3BGRL3-positive pixels that overlapped with mask pixels.

### Migration assays

Cells were plated in 6-wells plates (Corning) in DMEM containing 10% FBS, 1% Pen/Strep and 1% L-glutamine (Thermo Fisher). After 24 h from plating, siRNAs were transfected into the cells using Lipofectamine 2000 (Thermo Fisher), according to the manufacturer’s instructions. Forty-eight h after transfection, cells were starved using DMEM containing 1% Pen/Strep and 1% Glutamax without FBS. After 8–10 h of starvation, 25,000 cells were seeded in Boyden Chambers (Migration Support for 24 Well Plate with Transparent PET Membrane 8.0 μM pore in size, Corning). After 16 h of migration, cells were removed from the upper side of the filter with a cotton swab, and the cells on the lower side of the filter were stained using 1% Cristal Violet (Sigma-Aldrich) and fixed with 5% glutaraldehyde (Sigma-Aldrich). The lower side of the filters were photographed with a microscope, and cells were counted in double-blind with the aid of NIH Image software.

### Statistical analysis

Statistical significance of differences was evaluated by unpaired non parametric t-tests using the GraphPad Prism software (GraphPad Software Inc.).

## Supplementary Information

**Additional file 1.** Co-immunoprecipitation with an anti-FLAG-coupled resin from lysates of SKBR3 cells transfected either with FLAG-SH3BGRL3 or with the empty vector. Three bands indicated with an arrow were cut and further subjected to proteomic analysis. Results are reported in Additional File 2.

**Additional file 2.** Identification details of the proteins examined in this study. In the second sheet, protein identification details are given, such as protein band, protein accession and description, protein theoretical mass and pI values, protein score, protein matches, protein sequence coverage (%) and protein emPAI value. In the third sheet, peptide identification details are given, among which peptide rank, peptide experimental m/z value, peptide experimental Mr. value, peptide charge, peptide delta score, peptide score and peptide sequence.

## Data Availability

All data and plasmid constructs generated during the current study are available from the corresponding author on reasonable request.
