# Supplementary material for: SH3BGRL3 binds to myosin 1c in a calcium dependent manner and modulates migration in the MDA-MB-231 cell line
Source: BMC Mol Cell Biol. 2021 Aug 11;22:41. doi: 10.1186/s12860-021-00379-1 (PMC8356473; doi:10.1186/s12860-021-00379-1)
Supplement: Supplementary file 1 — Additional file 1. Co-immunoprecipitation with an anti-FLAG-coupled resin from lysates of SKBR3 cells transfected either with FLAG-SH3BGRL3 or with the empty vector. Three bands indicated with an arrow were cut and further subjected to proteomic analysis. Results are reported in Additional File 2. [file 12860_2021_379_MOESM1_ESM.pdf]

SH3BGRL3 binds to Myosin 1c in a calcium dependent manner and modulates migration in the MDA-MB-231 cell line

Filippo Di Pisa, Elisa Pesenti, Maria Bono, Andrea N Mazzarello, Cinzia Bernardi, Giovanni Renzone, Andrea Scaloni, Ermanno Ciccone, Franco Fais, Silvia Bruno, Paolo Scartezzini, Fabio Ghiotto

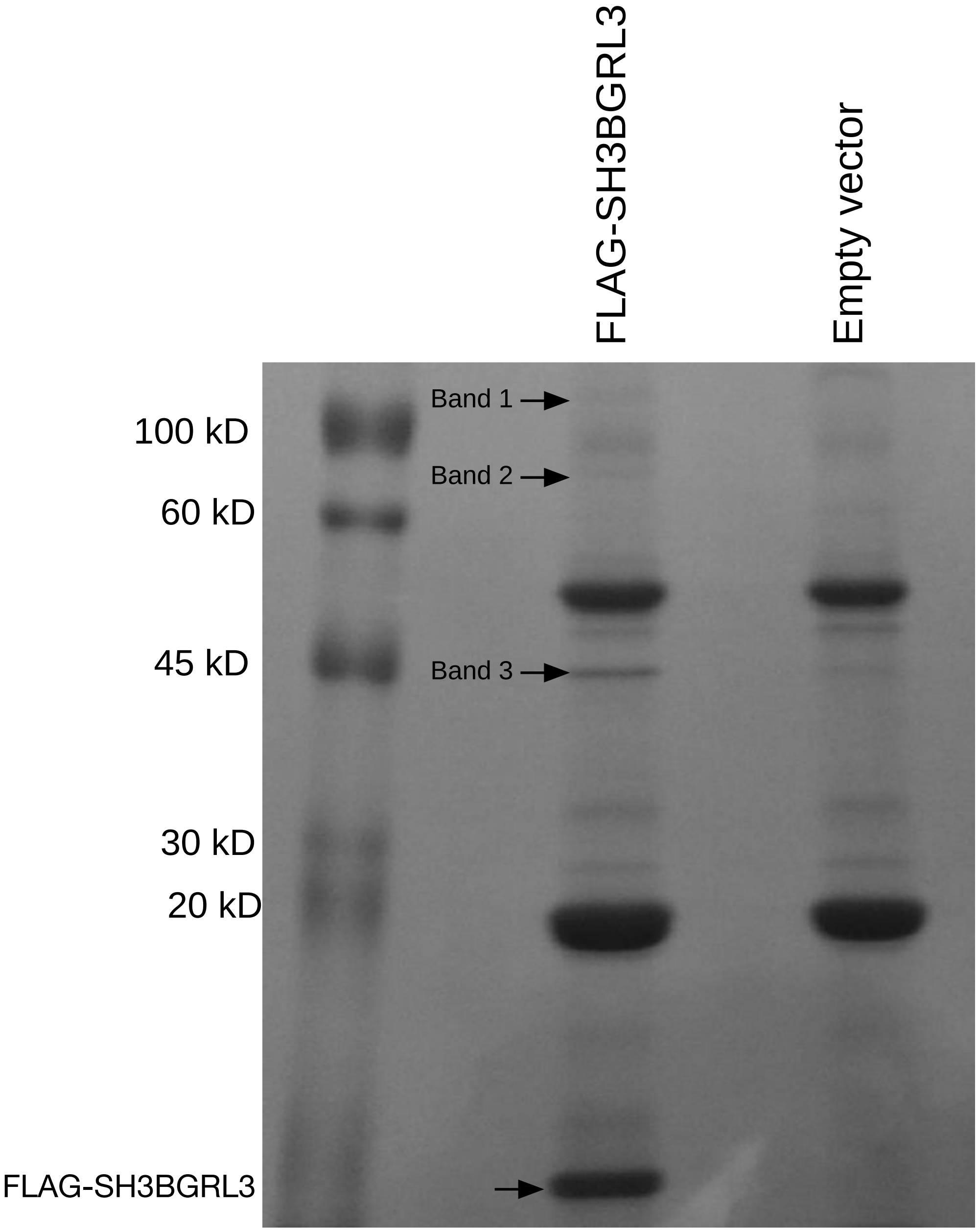

Additional File 1. Co-immunoprecipitation with an anti-FLAG-coupled resin from lysates of SKBR3 cells transfected either with FLAG-SH3BGRL3 or with the empty vector. Three bands indicated with an arrow were cut and further subjected to proteomic analysis. Results are reported in Additional File 2.
